# Association between maternal exposure to phthalates and lower language ability in offspring derived from hair metabolome analysis

**DOI:** 10.1038/s41598-018-24936-5

**Published:** 2018-04-30

**Authors:** Beatrix Jones, Ting-Li Han, Thibaut Delplancke, Elizabeth J. McKenzie, Jamie V. de Seymour, Mei Chien Chua, Kok Hian Tan, Philip N. Baker

**Affiliations:** 1 Department of Statistics, University of Auckland, 38 Princes Street, Auckland, 1010 New Zealand; 2 Liggins Institute, University of Auckland, 85 Park Road, Grafton, Auckland 1023 New Zealand; 3 First Affiliated Hospital of Chongqing Medical University, No. 1 Yixueyuan Road, Yuzhong District, Chongqing, 400016 China; 4 Department of Obstetrics & Gynaecology, Yong Loo Lin School of Medicine, National University of Singapore, NUHS Tower Block, Level 12,1E Kent Ridge Road, Singapore, 119228 Singapore; 5 Department of Obstetrics and Gynaecology, KK Women’s and Children’s Hospital, 100 Bukit Timah Road, Singapore, 229899 Singapore; 6 DUKE-NUS Medical School, 8 College Road, Singapore, 169857 Singapore; 7 College of Life Sciences, University of Leicester, University Road, Leicester, LE1 7RH United Kingdom

## Abstract

The fetus undergoes a crucial period of neurodevelopment in utero. The maternal hair metabolome provides an integrated record of the metabolic state of the mother prior to, and during pregnancy. We investigated whether variation in the maternal hair metabolome was associated with neurodevelopmental differences across infants. Maternal hair samples and infant neurocognitive assessments (using the Bayley III Scales of Infant Development at 24 months) were obtained for 373 infant-mother dyads between 26–28 weeks’ gestation from the Growing Up in Singapore Towards Healthy Outcomes cohort. The hair metabolome was analysed using gas chromatography-mass spectrometry. Intensity measurements were obtained for 276 compounds. After controlling for maternal education, ethnicity, and infant sex, associations between metabolites and expressive language skills were detected, but not for receptive language, cognitive or motor skills. The results confirm previous research associating higher levels of phthalates with lower language ability. In addition, scores were positively associated with a cluster of compounds, including adipic acid and medium-chain fatty acids. The data support associations between the maternal hair metabolome and neurodevelopmental processes of the fetus. The association between phthalates and lower language ability highlights a modifiable risk factor that warrants further investigation.

## Introduction

Between 10–20% of individuals worldwide have a neurodevelopmental disability^1^. During pregnancy, the fetus undergoes a period of intense neurodevelopment, making this a key time frame for understanding variability in cognitive traits. Poor maternal nutrition is a known risk factor for neurodevelopmental delay in the infant^2^, as are environmental exposures^3^. The maternal metabolite profile, referred to as the metabolome, consists of the low molecular weight compounds (<1 KDa), and is dictated by the interplay between environment (including food intake and environmental exposures), the microbiome, and host cellular processes. In particular, studies have shown the maternal metabolome to be highly influenced by maternal BMI^4^ and smoking^5^, as well as being responsive to the metabolic changes that occur in the development of pregnancy complications such as fetal growth restriction^6^ and preeclampsia^7^. Targeted metabolomics studies have found links between the maternal plasma and urine metabolomes and infants’ cognitive development^8,9^. Recently, maternal metabolomic profiling has expanded from analysis of typical biofluids such as blood and urine, and tissues such as placenta, to maternal hair. The maternal hair metabolome has been explored as a biological specimen to identify potential biomarkers and underlying metabolic mechanisms associated with the development of adverse pregnancy outcomes^10,11^. The hair metabolome has advantages over other more transient specimens such as urine and blood as it potentially offers a long term average of metabolite levels, and hair can be obtained non-invasively and stored easily. Pilot studies have suggested hair has a characteristic metabolite profile for type II diabetes^12^ and animal studies have shown nutrition affects the amino acid composition of animal hair^13^. However, no studies have been reported to investigate associations between the maternal hair metabolome and neurocognitive development of the offspring. Given the associations reported between the maternal urine and plasma metabolome and infants’ cognitive development, and the utility of the hair metabolome for identifying metabolomic markers of pregnancy disorders, the aim of our study was to investigate whether there are associations between the maternal hair metabolome and variation in the infant’s neurocognitive development.

In this paper we focus on a subset of individuals from the GUSTO (Growing Up in Singapore Toward healthy Outcomes) cohort^14^. We have used gas-chromatography–mass spectrometry (GC-MS) to measure the metabolome of maternal hair collected at 26–28 weeks of pregnancy. We then assess whether there are associations between this metabolome and variation in infant neurocognitive development at age two years, evaluated using the five subscores of the Bayley III Scales of Infant Development (BSID-III)^11,15^: cognition, receptive language, expressive language, fine motor, and gross motor.

## Results

### Socio-demographic characteristics

The socio-demographic characteristics of the 373 individuals included in this study that were significantly associated with one of the raw assessment scores are shown in Table 1. Ethnicity, sex of the child, age of the child at the time of cognitive assessment (measured in days), maternal education, gestational diabetes mellitus and smoking status were significantly associated with at least one cognitive outcome. Participant characteristics that were considered but were not found to be associated with any BSID-III raw scores were: maternal age, maternal BMI, gestational age at delivery (considered as a continuous variable, or as a dichotomous variable with categories before and after 37 weeks), and adjusted birth weight. Full results of all tests of association can be found in Supplementary Table 1. Many of the characteristics significantly associated with the cognitive scores were also associated with each other, so a more parsimonious model was constructed and used to control for socio-demographic characteristics in subsequent analyses. This model included ethnicity, sex of the child, age of the child at time of assessment, and maternal education. We note that explicitly controlling for the age of the child in days allows us to use the finer resolution raw score as a response.

**Table 1:** Participant charateristics significantly associated with at least one raw BSID-III score.

Participant Characteristics	Frequencies or Mean (sd)	P-values for associations with raw scores. For significant associations, Pearson’s correlation (continuous variables) or the category with the highest mean score is given.				
		Cognitive	Receptive Language	Expressive Language	Fine Motor	Gross Motor
Ethnicity	Chinese: 58% Malay: 26% Indian: 16%	0.31	**0.02** **Chinese**	0.71	0.40	<**0.01** **Malay**
Highest Level of Maternal education	University: 39% GCE A level: 34% Secondary: 22% Primary: 5%	<**0.01** **University**	<**0.01** **University**	<**0.01** **University**	<**0.01** **University**	0.13
Sex of Child	Male: 58%	0.20	**0.01** **Female**	**0.02** **Female**	0.15	0.23
Child’s age in days	733 (16)	**0.01** **r = 0.14**	0.07	0.11	**0.00** **r = 0.20**	0.99
Gestational Diabetes Mellitus (GDM)	Yes: 20%	**0.02** **GDM**	0.13	**0.02** **GDM**	**0.04** **GDM**	0.26
Smoking	Smoker: 40%	**0.03** **Nonsmoker**	<**0.01** **Nonsmoker**	0.09	0.24	0.83

Association p-values are based on Welch’s t-test for dichotomous variables, ANOVA for categorical or ordinal variables with more than two categories, and tests of correlation for continuous variables. Values less than 0.05 are shown in bold. For significant associations, Pearson’s correlation (continuous variables) or the category with the highest mean score is given.

### Metabolome

Our untargeted analysis provided intensity measurements for 276 metabolites; 182 of these have putative (library match >75%) or tentative (library match between 60–75%) identifications.

The single metabolite analyses showed associations between some metabolites and the expressive language scale, controlling for the socio-demographic variables. The expressive language responses showed an excess of metabolites with small p-values (5% of p-values are expected to be less than 0.05 when all null hypotheses are true; the observed value was 17%), and moderate q-values (see Table 2). Cook’s distance values were all less than 0.5, indicating that these relationships were not driven by outliers. Twenty-nine metabolites achieved our q-value criteria (q < 0.15) for inclusion in multiple regression analyses. Two of these were then excluded based on the missing value criteria, leaving a group of 27 for further consideration. Table 3 shows the identified metabolites from this group, annotated with the direction of associations with expressive language score. Retention times for unidentified metabolites meeting the criteria are given in Supplementary Table 2.

**Table 2:** Results of single metabolite analyses.

Scale:	Cognitive	Receptive language	Expressive language	Fine Motor	Gross Motor
% pval < 0.05	8%	7%	17%	3%	9%
Min p-value	0.008	0.010	0.003	0.003	0.004
Min q-value	0.28	0.48	0.15	0.59	0.37

**Table 3:** Identified metabolites achieving q < 0.15 for the relationship with expressive language, and meeting the missing value criteria.

Positively Associated with Expressive Language Score		Negatively Associated with Expressive Language Score	
Metabolite	Univariate p-value	Metabolite	Univariate p-value
*Alanine Derivative	0.003	p-tert-Butylbenzoic acid (PTBBA)	0.009
*2-Bornanamine, N-methyl, peak 1	0.012	Dipicolinic acid	0.016
**Adipic Acid**	0.014	Homoalanine	0.019
Stearic acid	0.016	*Orthoacetic acid	0.022
*Nonanoic acid*	0.017	**Phthalic Acid**	0.022
Salicylic acid	0.022	Methionine	0.024
*2-Bornanamine, N-methyl, peak 2	0.023	Alpha-ketobutyric acid	0.026
*Nudifloric Acid	0.023		
*Decanoic Acid*	0.026		

Asterisks denote tentative identifications (library match between 60% and 75%). P-values are from the single metabolite analyses controlling for demographic factors. Bolded compounds were selected in the multivariate model; other compounds correlated to these selected compounds with |r| > 0.5 are italicized.

### Multivariate predictor

The selected multivariate multiple regression model for expressive language had 6 predictors (sex of the child, maternal education, ethnicity, and three metabolites) and an R^2^ of 0.15. Coefficient estimates can be found in Supplementary Table 3. The metabolites selected (Adipic acid, Phthalic acid, and an unidentified metabolite with retention time 12.59) are shown in bold in Table 3 and Supplementary Table 3. The model selection process is expected to retain a low number of representatives from groups of interrelated metabolites; those with moderately high correlations (r ≥ 0.5) to the selected compounds are particularly important for interpretation and are italicized in the tables.

The receiver operating curve for predicting scaled expressive language score ≤5 had an area under the curve of 0.71. This represents a modest improvement over the prediction using only demographic variables (AUC 0.67).

## Discussion

With intensities for 276 compounds, we have measured a more extensive hair metabolite profile than previous studies^10–12^. The hair profile contained compounds that we expected based on what is known about hair composition^16^ and what types of compounds are detectable, given the choice of derivatisation method and analytical instrumentation. These comprised amino acids, benzenoids, carboxylic acids, fatty acids, hydroxy acids, and keto acids.

The raw expressive language scores from BSID-III have more and stronger associations with maternal hair metabolites than would be expected by chance, after adjusting for ethnicity, maternal education, exact infant age and sex. We note that we are examining ordinary variation in these scores, rather than disordered neurodevelopment; only the expected 2.5% (nine individuals) have their scaled expressive language scores <4 (i.e. more than two standard deviations below the mean using the BSID-III reference). The relationships observed provide intriguing links between the maternal hair metabolome and infants’ neurocognitive development, and merit further investigation. While a large number of associations (29) met the q-value criteria, we have restricted our focus to metabolites that either appeared in the multivariate predictor as representatives of the relevant processes. To aid in interpretation, we also consider compounds correlated with the metabolites in the multivariate predictor with |r| at least 0.50.

In the multivariate model, adipic acid represents a group of metabolites that are positively associated with expressive language score, meaning that higher levels of adipic acid and its associated group were associated with higher expressive language scores on the BSD-III. Excretion of adipic acid is associated with ketosis^17,18^ which can occur either when few carbohydrates are consumed, or when carbohydrate metabolism is impaired. Adipic acid is also included in controlled-release pharmaceuticals, antacids, and processed foods; it may be excreted when these are consumed^19^. In our sample, adipic acid is correlated at the 0.50 level with three compounds that also meet our q-value threshold: decanoic acid, nonanoic acid, and n-methyl-2-bornylamine. Decanoic acid is a major component of the medium chain triglyceride ketogenic anti-seizure diet; both decanoic and nonanoic acid have been shown to be effective for seizure control^20^. An investigation in *ex-vivo* rats has shown that the mechanism of seizure control for decanoic acid is inhibition of excitatory neurotransmission via AMPA glutamate receptor inhibition^21^. AMPA receptors have been implicated in neuronal injury resulting from excitotoxicity^22^, particularly in the early postnatal period^23^. The association of AMPA inhibitors, nonanoic and decanoic acid, with higher expressive language scores poses an interesting question about whether these fatty acids have a role supporting optimal neurodevelopment.

Our analysis shows a negative relationship between language scores and phthalic acid levels, meaning that higher levels of phthalic acid were associated with lower language scores on the BSD-III – the opposite direction to the relationship observed between adipic acid and expressive language scores. Many studies have found associations between phthalic acid and a variety of cognitive disorders^24^. Phthalic acid is an endocrine disruptor, with sex-differentiated neurocognitive effects. Two independent studies found adverse effects on motor reflexes in male infants^25,26^. However, another study of infants aged 2–3 showed that girls’ mental development index was negatively associated with phthalate exposure while boys’ psychomotor development index was positively associated with phthalate exposure^9^. Other studies have shown that elevated phthalates in maternal urine are negatively associated with motor development for two and three year old girls^5^, and are positively associated with behavioral problems in eight year olds^21^. Phthalates are thought to affect steroidogenesis in the sex organs^9,27,28^, and while they definitely affect rodent brain development in utero^29^, little is known about their effect in utero in humans, although widespread disruptions in hippocampal functional and structural plasticity are postulated^30,31^. Interestingly, a Polish study found that prenatal phthalate exposure was inversely associated with child psychomotor development, but postnatal exposure did not^32^, and an American study found attention deficit in children whose mothers had high prenatal levels of urinary phthalates^33^.

Phthalates are common compounds added to plastics to make them flexible. Phthalic acid in biosamples originates from exposure to plastics, via food packaging, particularly of hot foods and beverages, dermal exposure to cosmetics, and inhalation^34^. Acute exposure can occur from prolonged contact with flexible medical tubing and catheters due to hospitalization; these exposures have been associated with long-term attention deficit^35^. Elevated phthalate levels in dust from a child’s home have also previously been associated with lower verbal ability and developmental delay^36^. Phthalic acid was not correlated at the 0.50 level with any other known metabolites that met the q-value threshold.

The final compound in the multivariate model was an unknown with retention time 12.59 minutes. It is chemically similar to N-acetyl-1-methoxyethanimine (library match 56%). It did not have correlation 0.50 or larger with any known metabolites that met the q-value threshold.

While the non-invasive nature of hair sampling motivated the use of hair for this project, a shortcoming of our analysis was the use of full-length strands of hair. This means the metabolome measured was actually an average over the initial 26–28 weeks of pregnancy, as well as a variable period pre-pregnancy, depending on hair length. Future studies of the hair metabolome should consider performing segmental analysis, to allow the study of different time periods (e.g. pre-conception, first trimester, second trimester).

Another limitation was the cognitive assessment method. While BSID-III is widely used, it was developed in the United States and other researchers have found it necessary to establish new norms for different populations^37^. To check sensitivity to English language exposure, we considered a subset of our data (n = 257) where the children were given a score rating their exposure to English. This score was not associated with the expressive language score (p = 0.31), leading us to believe that the BSID-III was successfully administered in the Singaporean context. In addition, because we modelled the continuous scores rather than defining “low” or “high” categories, and adjusted for ethnicity in our models, we feel we have minimized the impact of cultural effects on our results.

It should also be taken into account that these findings are based on a cognitive assessment at a single time point. A conclusive finding of association between the maternal hair metabolome and the infants’ neurocognitive development would require tracking infants’ cognitive assessment over time. The findings from this study suggest such longitudinal tracking would be worthwhile.

In summary, the expressive language scores in the Bayley’s assessment of infant development for infants at 24 months of age are associated with levels of several metabolites in the maternal hair metabolome. These associations were not seen for the receptive language, cognitive or motor scores. As in previous studies, higher maternal phthalate exposure was associated with poorer outcomes. This finding highlights an early modifiable risk factor for compromised infant neurodevelopment that warrants further investigation.

## Methods

### Ethical Statement

The GUSTO study was approved by the National Health Care Group Domain Specific Review Board (reference D/09/021) and the SingHealth Centralized Institutional Review Board (reference 2009/280/D). Research was conducted according to the Declaration of Helsinki and all participants gave their written informed consent at recruitment.

### Study Participants

The study was conducted on a subset of women from the GUSTO cohort - a large mother-child cohort that was first established in Singapore in June 2009. Women were recruited in their first trimester of pregnancy from KK Women’s and Children’s Hospital (KKH) and National University Hospital (NUH). Recruitment was completed in September 2010 (n = 1,247). The cohort comprised three main ethnicities in the Singapore population (Chinese, Indian, and Malay). Of the 1,247 participants recruited in the GUSTO cohort, 373 mother-infant dyads had a neurodevelopmental assessment of the infant at two years of age, and a high quality maternal hair metabolome measurement.

### Cognitive assessment

The Bayley III Scales of Infant Development (BSID-III)^9^ were administered by trained assessors, fluent in the primary language of the child. We considered separately the five component scores: Cognitive, Receptive Language, Expressive Language, Fine Motor Skills and Gross Motor Skills. 365 children had all five scores, and 373 had at least one. Raw scores were used, with explicit modelling of the effect of the child’s age in days.

### Sample Preparation

Hair samples were collected at 26–28 weeks of gestation. Hair was cut from the occipital area, 0.5 cm from the scalp with scissors. Samples were stored at room temperature until analysis. The hair samples were processed as part of a larger group of 971 samples. The order of hair samples was randomised, with the design across experimental days balanced with respect to gestational diabetes, small for gestational age, and preterm birth. Cognitive score was not considered when randomising the samples.

Hair samples were washed in Milli-Q water and methanol (Merck, analytical grade) twice. Hair strands were cut into short sections and weighed, and 0.5 mg–5.5 mg of hair (~2–3 strands) was spiked with 20 µL of an internal standard mixture containing 10 mM of alanine-2,2,3,3-d_4_; 2 mM of hydroxyphenylalanine-3,3-d_2_; 10 mM of citric-2,2,4,4-d_4_ acid; 10 mM of phenyl-d_5_-alanine (Sigma-Aldrich). Samples below 0.5 mg in weight were not included for analysis. Hair samples were hydrolysed by incubation in 1 mL of 1 M potassium hydroxide at 54 °C for 18 h. After hydrolysis, the hair extracts were neutralised to pH 7 by dropwise addition of sulphuric acid. To remove salts and other residues, 1 mL of methanol (100%v/v) was added to the neutralised samples and centrifuged at 2000 g for 5 min to separate the supernatant from insoluble hair debris. The supernatants containing hair extracts were evaporated to dryness using a rotary evaporator (Savant SPS121P Speedvac, Thermo) at 37 °C for 16 h. Samples were derivatised using methyl chloroformate (MCF)^38^. Briefly, samples were re-suspended in 200 μL 1 M sodium hydroxide and transferred to a silanised glass tube. Methanol (167 µL) and pyridine (34 µL) were added. The derivatisation was initiated by adding 20 µL MCF followed by vortexing for 30 s, then a further 20 µL MCF was added, followed by 30 s of vortexing. To extract the derivisate, 400 µL chloroform was added, vortexed for 10 s and then 400 µL of 50 mM sodium bicarbonate was added and vortexed for an additional 10 s. The aqueous layer was discarded and the extract was dehydrated with anhydrous sodium sulphate before transferring to vials for GC-MS analysis.

The instrument used was an Agilent 7890B gas chromatograph coupled to an 5977 A single quadrupole mass spectrometer; settings are given in Table 4. Mass spectrometer calibration was carried out prior to analysis; the septum and liner were changed every ~200 injections. For each analytical batch of 18 samples, one solvent blank was run to measure instrument carryover, one alkane mix to measure instrument response, one reference standard mix to measure derivatisation efficiency, and one negative control to measure lab contamination. Five quality control samples (an aliquot pooled from every sample) were run throughout each batch to measure reproducibility.

**Table 4:** GCMS instrument parameters.

Carrier Gas	Instrument grade helium (99.999%)
Sample injection	Automated injection 1 µL
Injector liner	Deactivated glass split/splitless 4 mm ID straight single taper inlet liner packed with deactivated glass wool
Injector temperature	290 °C
Injector flow	Splitless, purge flow 25 mL/min, 1 min after injection
Column flow	1 mL/min, constant flow, column head pressure 9 psi
Column Type	Fused silica, 30 m × 250 μm id × 0.15 μm with 5 m guard column. Stationary phase of 86% dimethylpolysiloxane and 14% cyanoprophylphenyl
Thermal program, transfer line and source temperatures, solvent delay	45 °C for 2 min, increased by 9 °C/min^−1^ to 180 °C, held 5 min, then increased by 40 °C/min^−1^ to 220 °C, held for 5 min, then increased by 40 °C/min^−1^ to 240 °C, held for 11.5 min, increased by 40 °C/min^−1^ to 280 °C and held for 2 min. The interface temperature was 250 °C and the quadrupole temperature was 230 °C.
Ionization mode	Positive, 70 eV
Acquisition mode	Operated in scan mode; started after 5.5 min with mass range between 38–550 amu with scan time of 0.1 s.
Detection Threshold	50 ion counts

### GC-MS Data Extraction

The reference metabolite methyl chloroformate derivative mass spectral library developed by S. Villas-Boas (185 compounds) and the National Institute of Standards and Technology (NIST) 2014 EI library (242,477 compounds) were both used for compound identification. Using the NIST subset library method developed by E J McKenzie for the Automated Mass-spectral Deconvolution and Identification System (AMDIS), a subset of the NIST library was constructed using results from Chemstation Probability Based Matching integrator for a representative selection of quality control and sample data files. The Chemstation integration parameters were set to be sensitive to very low abundance compounds, and search-match parameters were expansive, with 10-15 compounds per feature included, in order to produce a comprehensive subset library of ~6000 compounds. This approach was used to circumvent limitations imposed by AMDIS on mass spectral library size.

The raw files from the GC-MS were converted into CDF/AIA format and then deconvoluted using AMDIS. An R-script^39^ was used to integrate peak intensities. The R-script returns a value for the retention time bin defined by AMDIS for a particular ion across all samples, and is thus more sensitive than AMDIS, and has fewer missing values, but slightly more false positives. Both retention time and mass spectrum were used to match compounds against the Villas-Boas reference metabolite library. Mass spectrum alone was used to match against the NIST14 subset library. Matches below 60% are labelled as “unknown”. Matches between 60% and 75% are labelled as “tentative”, those >75% are putative identifications.

The values used were the maximum height at the apex of the most intense ion for the compound peak. Data were checked against negative controls to identify and remove analytical contaminants. Peaks that were not integrated well using the automated method were integrated individually using the R package Metab^40^.

### Normalisation

Normalisation was performed using the 971 hair samples from study participants and 285 quality control samples. For each metabolite, the internal standard with the highest correlation *r* to that metabolite, in quality control samples, was used for normalisation, if *r* exceeded 0.85. Otherwise, internal standard normalisation was omitted. Samples were then normalised by biomass. Batch variation was addressed by median centering of the samples, specifically by multiplying the sample measurements for a particular day and metabolite by *M/m*, where *m* was the daily median and *M* the overall median for that metabolite. For a small number of days and metabolites, this created missing values if the median was a non-detection. These days were excluded for the study of that metabolite, and these metabolites were excluded from multi-metabolite analyses. For each metabolite, non-detections were replaced with 0.9 times the smallest non-zero value for that metabolite. Finally, values were log transformed.

### Statistical Analysis

Associations between the five raw BSID-III scores and the characteristics in Table 2 were examined with correlation for continuous characteristics, Welch’s t-test for dichotomous variables, and ANOVA for ordinal and categorical characteristics with more than two levels. From the characteristics with a significant (p < 0.05) association for any of the five scores, a model was constructed to control for demographic factors in the single-metabolite regression analyses. This model included exact infant age, to allow for use of the raw scores, and enough demographic factors that adding further demographic variables did not significantly improve the fit of the model.

For each neurodevelopmental scale, a regression model was fitted, with the score as the response, and the chosen participant characteristics, along with a single log metabolite intensity, as the predictors. The p-values for the metabolic predictors were tabulated in each case, and q-values (estimates of false discovery rate) were computed using the method of Storey and Tibshirani^41,42^, separately for each neurocognitive score type.

Where there was an excess of significantly associated metabolites, we assessed the ability of the metabolites with q < 0.15 to predict the BSID-III score using multivariate regression. We restricted our candidate predictors to the metabolites with complete information meeting the q-value criteria, and to participant characteristics in the selected demographic model for the neurodevelopmental scales. Only observations that were complete in all of these variables (365) were used for model selection. Backwards stepwise selection with AIC was used to produce a model, followed by stepwise pruning of predictors with the largest multivariate regression p-values until all p-values were less than 0.05. Using this model, receiver operator curves were produced for prediction of scaled expressive language score ≤5.

To understand the relationships between the metabolite candidate predictors for the multiple regression, a correlation matrix was computed. Where correlation with a metabolite included in the selected model exceeded 0.5, these metabolites were considered as a group for purposes of interpretation.

## Electronic supplementary material

Supplementary Material
