# Supplementary material for: Association between maternal exposure to phthalates and lower language ability in offspring derived from hair metabolome analysis
Source: Sci Rep. 2018 Apr 30;8:6745. doi: 10.1038/s41598-018-24936-5 (PMC5928220; doi:10.1038/s41598-018-24936-5)
Supplement: Supplementary file 1 — Supplementary Material [file 41598_2018_24936_MOESM1_ESM.pdf]

Supplementary Material

Association between maternal exposure to phthalates and lower language ability in offspring derived from hair metabolome analysis.

Beatrix Jones PhD [1], **Ting-Li Han** PhD [2,3], **Thibaut Delplancke** PhD [3], **Elizabeth J McKenzie** PhD [2], **Jamie V de Seymour** PhD[2*], **Mei Chien Chua** MRCP (Paeds) [4,5,6], **Kok Hian Tan** MMed (O&G) [5], **Philip N Baker** DM [2,3,7]

1. Department of Statistics, University of Auckland, 38 Princes Street, Auckland 1010, New Zealand;
2. Liggins Institute, University of Auckland, 85 Park Road, Grafton, Auckland 1023, New Zealand;
3. First Affiliated Hospital of Chongqing Medical University, No.1 Yixueyuan Road, Yuzhong District, Chongqing 400016, China;
4. Department of Obstetrics & Gynaecology, Yong Loo Lin School of Medicine, National University of Singapore, NUHS Tower Block, Level 12,1E Kent Ridge Road, Singapore 119228, Singapore;
5. Department of Obstetrics and Gynaecology, KK Women's and Children's Hospital, 100 Bukit Timah Road, Singapore 229899, Singapore;
6. DUKE-NUS Medical School, 8 College Road, Singapore 169857, Singapore;
7. College of Medicine, Biological Sciences, and Psychology, Maurice Shock Building, University of Leicester, University Road, Leicester LE1 7RH, United Kingdom

Supplementary Table 1: Tests of association between participant charateristics and raw BSID-III score. Association p-values, test statistics, and degrees of freedom are based on Welch's t-test for binary variables, ANOVA for categorical variables with more than two categories, and tests of correlation for continuous variables. Cells for significant tests (p< 0·05) are shown in bold

Participant Characteristics	Frequencies or Mean (sd)	p-value for association with raw score; and r, or F with degrees of freedom. For F test with p< 0.05, the category with the highest score is indicated.				
		Cognitive	Receptive Language	Expressive Language	Fine Motor	Gross Motor
Ethnicity	Chinese: 58%<br>Malay: 26%<br>Indian: 16%	0.31<br>$F_{2,366}$=1.18	**0.02**<br>**$F_{2,365}$=4.18**<br>**Chinese**	0.71<br>$F_{2,364}$=0.37	0.40<br>$F_{2,362}$=0.93	**<0.01**<br>**$F_{2,360}$=7.22**<br>**Malay**
Maternal age at recruitment (years)	31.1 (4.8)	0.19<br>r=0.07	0.14<br>r=0.08	0.65<br>r=0.02	0.78<br>r=0.01	0.35<br>r=-0.05
Highest Level of Maternal education	University: 39%<br>GCE A level: 34%<br>Secondary: 22%<br>Primary: 5%	**<0.01**<br>**$F_{3,365}$=9.92**<br>**University**	**<0.01**<br>**$F_{3,363}$=17.40**<br>**University**	**<0.01**<br>**$F_{3,362}$= 10.02**<br>**University**	**<0.01**<br>**$F_{3,361}$= 4.55**<br>**University**	0.13<br>$F_{3,361}$= 1.90
Maternal BMI (first trimester)	25.9 (4.6)	0.53<br>r=-0.03	0.06<br>r=-0.10	0.46<br>r=-0.03	0.20<br>r=-0.07	0.26<br>r=-0.06
Sex of Child	58% Male	0.20<br>T_{363}=-1.29	**0.01**<br>**T_{366}=-2.59**<br>**Female**	**0.02**<br>**T_{355}=-2.42**<br>**Female**	0.15<br>T_{340} =-1.44	0.23<br>T_{362} =1.43
Gestational Age at Delivery (weeks)	38.7 (1.3)	0.64<br>r=-0.02	0.84<br>r=0.01	0.47<br>r=0.04	0.60<br>r=0.03	0.97<br>r=0.00
Premature (before 37 weeks)		0.13<br>T_{34}=-1.54	0.08<br>T_{29}=-1.82	0.80<br>T_{28}=0.26	0.27<br>T_{28}=-1.13	0.82<br>T_{30}=0.23
Child's age in days	733 (16)	**0.01**<br>**r=0.14**	0.07<br>r=0.09	0.11<br>r=0.08	**0.00**<br>**r=0.20**	0.99<br>r=0.00
Adjusted Birth weight (grams)	3122 (345)	0.69<br>r=-0.02	0.06<br>r= -0.10	0.70<br>r=-0.02	0.69<br>r=-0.02	0.20<br>r=-0.07
Gestational Diabetes Mellitus	GDM: 20%	0.02<br>T_{115}=-2.29<br>**GDM**	0.13<br>T_{117}=-1.53	0.02<br>T_{106}=-2.36<br>**GDM**	0.04<br>T_{118}=-2.03<br>**GDM**	0.26<br>T_{132}=1.13
Smoking	Smoke: 40%	0.03<br>T_{300}=2.13<br>**Nonsmoker**	< 0.01<br>T_{328}=3.68<br>**Nonsmoker**	0.09<br>T_{319}=1.69	0.24<br>T_{322}=1.17	0.83<br>T_{306}=0.22

Supplementary Table 2: Mass and retention time for unidentified compounds achieving the minimum q-value. Bolded compound appears in the Multivariate predictor.

Retention Time (minutes)	Direction of association	p-value
**12.59**	**neg**	**0.019**
15.24	neg	0.021
15.80	neg	0.021
15.78	neg	0.017
17.01	pos	0.023
17.58	neg	0.019
18.68	neg	0.016
23.89	neg	0.014
29.83	pos	0.014
29.98	neg	0.018
31.81	neg	0.009

Supplementary Table 3: Selected Multi-metabolite Model. Response is raw expressive language score. Metabolite predictors (bold) are on the natural log scale; the coefficient reflects the effect of a 1 standard deviation change.

Predictor	Coefficient	p-value
Intercept (Male Sex, Highest Maternal Education Primary, Chinese Ethnicity)	27.96	<0.001
Highest Maternal Education Secondary	1.99	0.144
Highest Maternal Education A level	2.92	0.027
Maternal Education University	5.55	<0.001
Malay Ethnicity	1.85	0.008
Indian Ethnicity	-0.59	0.442
Female Sex	1.72	0.002
**Adipic Acid**	**0.62**	**0.025**
**Unknown (Retention time 12.59m)**	**-0.57**	**0.038**
**Phthalic Acid**	**-0.59**	**0.032**
